# Comparison of Physical and Compositional Attributes between Commercial Plant-Based and Dairy Yogurts

**DOI:** 10.3390/foods13070984

**Published:** 2024-03-23

**Authors:** Likhitha Marlapati, Rabia F. S. Basha, Amelia Navarre, Amanda J. Kinchla, Alissa A. Nolden

**Affiliations:** Department of Food Science, University of Massachusetts Amherst, Amherst, MA 01003, USAkinchla@umass.edu (A.J.K.)

**Keywords:** quality, yogurt-like, texture, rheology, physicochemical

## Abstract

A primary strategy led by the food industry to improve the sustainability of the agricultural food supply is the development of modern plant-based alternatives. The information provided via marketing and product packaging provides consumers with the expectation that these products provide a similar product experience to conventional products, yet it is not well understood whether these commercial alternative products are comparable to traditional animal-based products. To aid in developing improved plant-based products, this study aimed to compare the quality and physical attributes of commercially available plant-based and dairy yogurts. Using instrumental methods, commercially available yogurt products were analyzed for their pH, titratable acidity, color, water activity, moisture content, and rheology, which included 13 plant-based (almond, cashew, coconut, oat, soy) and 8 whole-milk dairy yogurts. The present study reveals that the plant-based and dairy yogurts had no significant differences in pH, lactic acid, water activity, or moisture content. However, there were significant differences in the color and texture properties between the plant-based and dairy yogurts. Additionally, significant differences were observed across the plant-based yogurt products in terms of their color and texture properties. This highlights the need for additional studies to determine how individual ingredients influence the physical characteristics and textural properties to direct the development of plant-based yogurts. Improving upon the physicochemical properties of plant-based yogurt may encourage more consumers to adopt a more sustainable diet.

## 1. Introduction

Across the globe, the food industry is preparing to adapt to the future food supply, which will need to produce food for roughly 10 billion people by the year 2050 [1]. While the agricultural food sector faces multiple challenges to ensure a safe and nutritious food supply, there is growing concern about the sustainability of food production. There is focused interest in creating alternative low-carbon protein sources to meet the rising global demand for food and considering the environmental sustainability of food production [2,3,4]. Many alternative protein sources are available (e.g., tofu), yet these products have not been widely adopted by consumers [2]. The food industry has primarily focused on developing sustainable alternatives that utilize plant-based proteins and ingredients to recreate the functional and sensory properties typically found in animal-based products [5,6]. These alternatives are often called novel plant-based foods, imitation foods, or mimics. The plant-based market is estimated to be worth $28 billion as of 2022 [7]. While all plant-based product categories are expected to continue to grow, the biggest category achievements have been within the plant-based dairy category. 

As of 2022, the plant-based milk industry was valued at $2.8 billion, accounting for 35% of all plant-based sales [7]. In terms of all milk sales, plant-based milk accounts for 15% of total sales, demonstrating the potential of the impact of plant-based products [7]. In 2019–2022, there was a 19% increase in plant-based milk sales and, at the same time, a decline in dairy milk sales (4%) [7]. This success could be viewed as plant-based milk successfully transitioning consumers to a more sustainable alternative while demonstrating the potential within other plant-based dairy categories, such as plant-based yogurt. The U.S. market for plant-based yogurt is estimated at $425M [7]. This suggests that plant-based dairy has the potential to be integrated and adopted by consumers; however, plant-based yogurt has not seen the same success in terms of commercial sales compared to plant-based milk [7,8,9]. Therefore, this highlights the opportunity to compare the performance of commercially available plant-based yogurts to investigate potential areas of improvement.

When formulating a modern plant-based product, there are many ways to evaluate its performance and functionality to ensure similar characteristics to conventional products [10,11]. Examining the differences in its physicochemical structure and comparing its performance to conventional and plant-based products (e.g., soy vs. almond) help identify opportunities to improve its functionality as a primary strategy to assess the overall success of different formulations. Of particular interest are the physicochemical attributes that have a role in producing mouthfeel characteristics and correspond to consumer liking. For example, Greis and colleagues (2020) identified that commercial plant-based oat yogurts were perceived as thin and watery, which was negatively associated with liking [12], which is consistent with other findings [13]. A recent study reported that among a cohort of consumers in New Zealand, commercially available plant-based yogurts had low acceptability, driven by high perceptions of sourness and an undesirable appearance (lumpy and non-white) [14]. These results concluded that the discrepancy between the expected and actual sensory profiles was the primary driver of the rejection of these products. These studies suggest functionality differences between plant-based and dairy yogurts warrant a more comprehensive investigation across a wide variety of commercially available plant-based yogurts. 

The evidence demonstrates that the textural properties of plant-based yogurt are driven by the ingredients and processing steps [8,15]. From a formulation standpoint, these textural differences are partially due to plant-based yogurts lacking the casein and lactose molecules found in milk, making it a challenge to recreate the traditional gel network formed in dairy yogurt. In the absence of stabilizers, the gelation process is a key component in controlling the textural properties of the finished yogurt product [16]. Compared to animal-based systems, plant-based components have lower inherent gelling strengths; hence, hydrocolloids are used to improve the gelling structures [17]. However, instrumental texture measurement is not the only physical characteristic that is important to measure when assessing performance and functionality. Physical properties such as water activity influence acid production, and subsequently, pH is vital for the growth and viability of the microorganisms responsible for yogurt formation [15,18].

Previous studies have demonstrated differences in the physical characteristics of commercially available plant-based yogurts [19,20,21,22]. Grasso and colleagues (2020) examined six commercially available plant-based yogurts available in Ireland and determined that some plant-based products performed more similarly to dairy yogurts than others. For example, soy, coconut, and cashew were more comparable to dairy than hemp and almond in terms of their textural properties [19]. While this study provides new insights into comparing plant-based and dairy yogurts regarding their physicochemical properties, it has limited ability to generalize across plant-protein yogurts, as the analysis was conducted for one sample per base yogurt (e.g., oat, soy, pea). These findings were further supported by Wang and colleagues (2023) with yogurts prepared in a laboratory setting [21]. These instrumental methods remain the primary method for measuring yogurt performance, at least in experimental research settings [23,24]. Examining these differences provides the food industry with an insight into the limitations of improving and opportunities to improve the functional characteristics of plant-based yogurts, which helps to drive greater consumer acceptance. 

This study aims to characterize the physicochemical and rheological properties of commercial plant-based and dairy yogurts to gain insight into the opportunities for the food industry to focus on characteristics dissimilar to that of conventional dairy yogurt. Here, instrumental analysis was conducted to measure the pH, water activity (Aw), moisture content, color, titratable acidity, and rheology of diverse yogurts made from different protein bases. This new knowledge expands on past work and provides new insights into the US product market for plant-based yogurt to support the development of plant-based yogurts that provide a similar eating experience to dairy yogurt. 

## 2. Materials and Methods

### 2.1. Samples

Commercial yogurts (dairy and plant-based) in the United States were purchased in grocery stores in Amherst, MA, and surrounding towns. The retail stores included Walmart, Target, ALDI, Whole Foods Market, Stop & Shop, Big Y, and Trader Joe’s. A total of 21 yogurts were purchased, with 8 whole-fat dairy yogurts and 13 plant-based with different plant-protein bases: almond (n = 4), cashew (n = 2), coconut (n = 4), oat (n = 2), soy (n = 1). Each yogurt base’s average caloric, fat, and protein contents are reported in Table 1, along with the standard deviation of each component. A list of products and their ingredients can be found in Supplemental Table S1. 

All the yogurts were either vanilla-flavored or plain to eliminate potential variability in the physical characteristics due to different flavors or add-ins. We found that whole-milk dairy yogurts contained a similar fat content to the plant-based yogurts; therefore, we only included whole-milk and full-fat dairy yogurts for this study. Greek dairy yogurts were excluded due to their high protein content and the lack of available plant-based alternative equivalents on the market.

### 2.2. pH

The pH of the yogurt samples was measured using a pH meter (Oakton pH 6+ Handheld, Hanna Instruments model HI1131, Smithfield, RI, USA) at 24 °C. The instrument was calibrated using calibration standards (pHs of 4, 7, and 10).

### 2.3. Titratable Acidity

The titratable acidity followed AOAC method no. 947.05 [24]. Briefly, the percent lactic acid of the yogurt samples was measured by neutralizing the yogurt samples with NaOH titrant solution using an automatic dairy titrator (Hanna Instruments HI84529U and HI1131, RI, USA). A 3-point calibration procedure was followed using calibration standards of pHs of 4.01, 8.30, and 10.00. Each sample (20 mL) was diluted with 40 mL of deionized water and titrated using a High Range 20 titrant (HI84529-51) until the endpoint was reached.

### 2.4. Color

The optical properties of the samples were quantified using an instrumental colorimeter (ColorFlex EZ 45/0-LAV, Hunter Associates Laboratory Inc., Reston, VA, USA). The L* value refers to brightness, ranging from 0 (black) to 100 (white). The a* value indicates the degree of redness (positive values) or greenness (negative values), whereas the b* value measures the degree of yellowness (positive values) or blueness (negative values). The Whiteness Index was calculated using Equation (1), given below. Initially, the instrument was calibrated before the samples were analyzed against a white tile background.

Whiteness Index = 100 − [((100 − L*)^2^ + a*^2^ + b*^2^) ^(1/2)^] (1)

### 2.5. Water Activity

The water activity of each yogurt sample was measured using a Decagon Devices water activity meter (AQUALAB 4TE, Pullman, WA, USA).

### 2.6. Moisture Content Analysis

The moisture content was measured using a halogen moisture analyzer (MX-50, A&D Company, Tokyo, Japan). Each sample (3 g) was placed in a metal tin and heated to 105 °C. The weight of the sample, % moisture, and runtime were recorded.

### 2.7. Rheology

The rheological properties of the yogurt sample were measured using a TA HR 20 Discovery rheometer (TA Instruments, New Castle, DE, USA). Each sample (20 mL) was cooled to 10 °C in a Peltier concentric cylinder, and a conical rotor was used to determine the sample’s shear stress (σ) in Pa. An increasing shear rate (ƞapp) from 0 to 200 s^−1^ was applied for 200 s, followed by a decreasing shear rate from 200 to 0 s^−1^, also applied for 200 s [13]. Hysteresis curves were developed for each sample to compare the rheological differences between the yogurt bases. The flow behavior index (n) and consistency coefficient (K) were calculated using the power law equation (Equation (2)):

σ = Kγ^n^ (2)

The dependence of the viscosity on the shear rate for all the samples was fitted to the Ostwald–de Waele equation (Equation (3)), previously applied to yogurt [25], where η_app_, K, γ, and n refer to the apparent viscosity (Pa.s), the consistency index (Pa.sn), the shear rate (1/s), and the flow behavior index (dimensionless), respectively:

ƞ_app_ = Kxγ ^(n−1)^ (3)

### 2.8. Statistical Data Analysis

Each measurement was carried out in triplicate, with three commercial yogurt samples measured individually. The data for all the parameters measured were first checked for normality. The data were not normally distributed; hence, the Analysis of Variance (ANOVA) test was employed to conclude the equality of the medians between the samples. The post hoc Tukey’s Honest Significant Differences (HSD) test was used to identify significant pairs, defining significant differences between the samples (*p* < 0.05). All the analyses used R (R Core Team 2021, version 4.1.2, R Foundation for Statistical Computing, Vienna, Austria).

## 3. Results

### 3.1. Nutritional Composition

The nutritional content of the yogurts (energy, fat, protein, and carbohydrates) is reported in Table 1. All the nutrients are standardized per 100 g. There were no significant differences in the total energy (Kcal) or the fat and carbohydrate content across all the product bases. The total energy per 100 g of yogurt was between 76.8 and 101.7 (Kcal), with almond yogurt having the highest energy and coconut yogurt having the lowest energy. The fat content ranged from 2.3 to 5.5 g, with almond yogurt having the highest fat content and soy yogurt having the lowest fat content. The carbohydrate content ranged from 9.0 to 12.8 g, with oat yogurt having the highest and dairy having the lowest. The protein content ranged from 0.2 to 4.0 g, with coconut having the lowest and soy having the highest. There were significant differences in the protein content, with coconut having significantly lower protein (0.2 g) than all the other products, with the exception of cashew (2.0 g). All the products had a fiber content of less than 1.7 g. 

### 3.2. pH and Titratable Acidity

The pH and TA values are presented in Table 2. Across the products, the pH values ranged between 3.7 and 4.8. The statistical analysis revealed no significant differences in the pH between the dairy yogurt and all the other plant-based yogurts. However, there were significant differences in the plant-based yogurts, with cashews and oats significantly lower than almonds and soy. The TA of the yogurts ranged between 0.3% and 0.9% (Table 2), revealing that coconut (0.3%) and oat (0.3%) were significantly different from dairy (0.9%). Still, no significant differences were identified between soy, almond, cashew, and dairy (Table 2). 

### 3.3. Color

The results of the color analysis are presented in Table 3. For brightness (L*), dairy yogurt had the highest (91.9), and oat had the lowest (78.2). When comparing all the plant-based yogurts to dairy, only soy and cashew had L* values that were statistically similar to dairy. For the coordinates associated with the presence of green and red colors, dairy exhibited the most negative value (a* at −1.1), indicating the presence of green, while oat showed the most positive value (1.6), indicating the presence of red. Further analysis revealed that cashew, coconut, and soy were not statistically different from dairy yogurt (Table 3). For b* (the coordinates responsible for the presence of the colors blue/yellow), soy had the highest value (17.3), and coconut had the lowest value (7.5), with oat being the only plant-based yogurt significantly different from dairy. The Whiteness Index (WI) of dairy and coconut yogurt had no significant difference. The oat yogurt (72.3) significantly differed from the dairy and other plant-based yogurts. Dairy had the highest WI (86.46), followed by coconut, cashew, soy, almond, and oats.

### 3.4. Moisture Content and Water Activity

The results for the moisture content (MC) and water activity (Aw) analyses reported no significant differences between product categories (*p* > 0.05). The MC values ranged between 53.8 and 58.2. The Aw ranged between 0.97 and 0.99, with no significant differences observed between the dairy and plant-based yogurts. 

### 3.5. Rheological Properties

The rheological properties were examined and compared for differences in the flow behavior index (n), consistency coefficient (k), R^2^, apparent viscosity, and area in the loop (HLA) of the plant-based and dairy yogurts (Table 4). The calculated R^2^ is used to assess the fit, consistency coefficient (K), and flow behavior index (n) values using the Power Law. The apparent viscosity (ƞapp) was calculated using the Ostwald–de Waele equation. The area in the hysteresis loops (HLAs) were measured to determine the thixotropic properties of the yogurts. The hysteresis loop (the area between the upward and downward curves) measures the extent of structural breakdown during shearing. 

**Figure 1:**
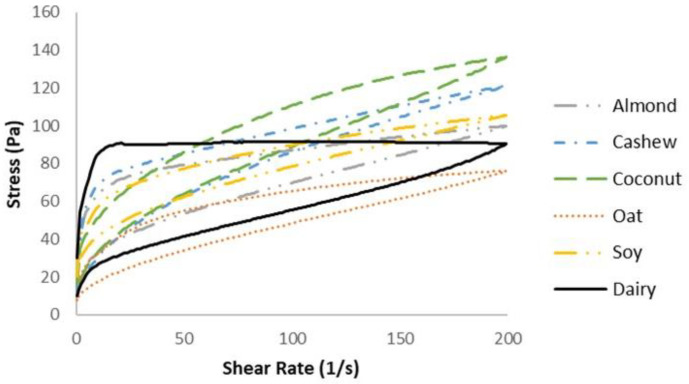
The hysteresis loops of yogurt samples. Comparative shear rate–stress plots for yogurts as a function of increasing shear rate from 0 to 200 s^−1^ and reducing shear rate from 200 to 0 s^−1^ at 10 °C.

The yogurts showed no significant difference for R^2^, K, n, and ƞapp but significantly differed in the HLA. All the yogurts showed shear-thinning (n < 1) behavior, indicating that the apparent viscosity decreased as the shear rate increased. The dairy yogurts had a significantly greater area in the loop than all the plant-based yogurts (Figure 1 and Table 4). 

## 4. Discussion

### 4.1. Nutritional Composition

While alternatives to traditional dairy yogurt derived from plants are growing in popularity, questions about their nutritional content persist [26,27]. The preliminary data suggest there are nutritional concerns when diets substitute conventional products with novel plant-based ones [28,29,30]. Within plant-based dairy, prior work has demonstrated significantly less protein compared to conventional dairy [26,31,32]. Comparatively, in this study, of all the plant-based yogurts studied in this work, soy had the highest protein content (4.0 g/100 g), followed by dairy (3.6 g/100 g). According to Clegg and colleagues (2021), soy yogurt had a protein content more than twice that of nut-based yogurts and the lowest fat content [31]. Recent findings confirm these results, which demonstrates the variability in the protein content across plant-based yogurts [33]. The results from the present study align with the available literature suggesting plant-based yogurts may provide similar amounts of protein, except for coconut, since no significant differences were observed. While soy had the highest protein content, it also had the lowest fat content (2.3 g). Even though, in the present study, there were no significant differences in fat or energy (Kcal), prior studies report significant differences in the fat and energy of plant-based yogurt [19,34]. The research conducted by D’Andrea et al., 2023, concluded that while plant-based yogurts have more fiber, less sodium, and less total sugar than dairy yogurts, they also have less protein, calcium, and potassium [26]. Using a comprehensive nutrition score, which considers multiple macro and micronutrients, it determined that almonds have the highest nutritional density, followed by dairy and oat yogurts. Furthermore, the range in values for the nutritional composition is comparable in the present study with the comprehensive nutritional assessment completed by D’Andrea and colleagues (2023). Strategies to improve the nutritional profiles can include fortifying plant-based yogurts with the required nutrients [26]; using blended plant-based milk to produce yogurts [15,35], where the kinds of milk are selected in such a way that they make up for each other’s deficiencies and have nutritional profiles similar to dairy; and also investigating other novel fermented plant-based milks that might have properties and nutritional profiles similar to dairy yogurt.

The nutritional differences extend to other plant-based categories [29,36], such as beef [37,38], cheese [39,40], and milk [31,41]. With this growing support for concerns about the nutritional differences in plant-based foods, the food industry should consider future research to investigate new approaches, such as fermentation, to improve their nutritional profile [42,43] and create plant-based products with similar dietary profiles if consumers intend to replace conventional products with plant-based alternatives. 

### 4.2. The Differences in the Physicochemical Attributes of Plant-Based and Dairy Yogurts

#### 4.2.1. pH and Titratable Acidity

pH plays a crucial quality control step in the manufacturing of yogurt, assessing the acid development of a dairy product and typically identifying the fermentation process’s endpoint [44]. It also serves as an indicator that contamination by bacteria or chemicals has occurred. The pH values varied amongst the plant-based yogurts but were not significantly different from dairy yogurt. This was expected due to the addition of acid regulators like citric acid, malic acid, and tricalcium citrate identified in the ingredient list of the plant-based yogurts. Grasso and colleagues, 2020, suggested that the reason for having a wide range of pH values among plant-based yogurts is to optimize the activity of various gelling agents [19]. These added thickeners affect the viscosity of the yogurt, as the addition of hydrocolloids likely contributes to the viscoelastic properties of yogurts [45]. 

Lactic acid is responsible for giving yogurts their structure, texture, and sensory attributes. The lactic acid content of the plant-based yogurts was significantly lower than that of the dairy yogurts. As suggested previously, this is likely because yogurt made from plants does not include lactose, so the bacteria cannot form enough lactic acid to help give yogurt its texture [46]. Additional thickeners and stabilizers are often used in plant-based yogurts to overcome the negative impact of lower amounts of lactic acid on the structure. 

#### 4.2.2. Color, Moisture Content, and Water Activity

The product’s appearance or color partially influences the consumer acceptability of yogurt [46]. Protein and fat globules’ capacity to scatter and reflect light are connected to yogurt’s brightness, and the size of these molecules is significantly determined by the processing methods and unit operations used [19]. Regarding brightness (L*), cashew and soy were most similar to dairy. However, soy had the highest b* value and the most negative a* value after dairy, confirming the prior work by Tangyu et al., 2019, reporting a more greenish, grayish, or brownish color associated with various types of plant-based milk [42].

The taste, appearance, texture, and shelf life of yogurt are influenced by its moisture content (MC). It is important to maintain the MC to maintain consistency and microbial growth. A possible reason for the high MC in coconut yogurt could be coconut’s very low protein content. This would align with soy having the lowest MC and the highest protein content. Adding various gels and hydrocolloids to plant-based yogurts would also affect their MC. A key indicator of microbial development in yogurts is water activity (Aw). A study by Lucatto and colleagues, 2020, found the water activity of dairy yogurt to be between 0.97 and 0.99 [47]. The observations from the present study align with the literature, with the Aw ranging from 0.980 to 0.991. This shows that the ideal water activity is achievable irrespective of the base used. The water activity is not affected in plant-based yogurts.

### 4.3. Rheological Properties

The flow behavior index (n) values of all the yogurts demonstrated shear-thinning behavior (n < 1). The dairy-based yogurts had a visibly larger hysteresis loop area (HLA), confirmed using the area calculation under the curve (Table 4). Thixotropic behavior is defined by a loss of structural strength during the shear phase; however, there is a complete structural recovery once the shear is removed. A larger HLA value indicates a weaker thixotropic behavior, meaning a slow structure recovery. It is established that dairy-based yogurts have a low structure recovery, which affects their apparent viscosity [48]. However, compared to the dairy yogurts, the plant-based products had noticeable and much smaller hysteresis loop regions, suggesting a quicker structural repair rate, indicating a thinner textural consistency. As previously suggested, this is caused by the variations in the gelling agents and how they interact within the yogurt matrix [45].

One study found that the hysteresis loops were the largest in soy/coconut yogurts with a higher percentage of coconut milk, meaning their thixotropic properties decreased with an increase in coconut milk concentration [49]. This aligns with the current findings that the soy sample had a smaller HLA than coconut. Another study found that the almond sample had very strong thixotropic behavior [19], contrasting the present findings, where it had a similar HLA to coconut. In this sense, coconut and almond are most similar to the dairy samples due to their poor structural reversibility. Kosterina and colleagues, 2020, also found that coconut milk increased the viscosity of yogurt, relating the thixotropic behaviors to the apparent viscosity [49]. This aligns with our data since the coconut samples had the highest maximum stress, indicating higher resistance to shear forces and ‘firmness.’

### 4.4. Limitations and Looking to the Future of Plant-Based Yogurt 

This study identified 13 commercially available plant-based yogurts. However, only one soy yogurt was identified due to its limited availability. Additional studies consisting of more soy-based yogurts may reveal variations between products. To reduce waste during analysis, after the textural analysis was conducted, the samples were frozen to preserve their quality; however, it is possible that freezing may have influenced product features. Yet, all products were frozen under the same conditions, and this would have impacted all products similarly. 

This study highlights the quality and textural differences between commercial plant-based and dairy-based yogurts. Plant-based yogurts produced from raw plant-based milk form a gel, but compared to dairy, they form weak gel networks. They are known to exhibit shear-thinning properties [50]. The viscosity of raw plant-based yogurts is significantly lower than that of dairy. Hence, hydrocolloids are added to improve their viscosity and gel strength [19]. Among plant-based milks, soy milk had a higher viscosity and gel strength than other plant-based yogurts [50]. Fermented soy protein isolates have been explored for application in plant-based yogurts and found to have a viscosity and gel strength similar to dairy [51]. 

Another potential solution for addressing the functional differences in these yogurts is creating hybrid or blended yogurts, i.e., yogurt made by combining dairy milk and plant protein. Several studies suggest the benefits of mixing plant protein with dairy yogurt, resulting in more nutritious and well-textured yogurt [52] and potentially improving consumer acceptance and mouthfeel attributes [35,52]. This approach has been considered for other dairy categories, such as cheese, to improve the physicochemical properties and sensory profile [53]; yet, challenges continue to exist in the acceptance of hybrid alternatives [54,55]. However, no hybrid products were identified in our search, and future studies should examine the potential advantages of hybrid yogurt products in terms of their physical properties and consumer acceptance. 

## 5. Conclusions

A primary strategy of modern plant-based alternatives is to create products that mimic conventional animal products. This study aims to contribute to the scientific field of plant-based alternatives by investigating whether the current commercial plant-based yogurts match the functional performance of traditional dairy yogurts. Here, we demonstrate the differences in the physical characteristics of dairy and plant-based yogurts and highlight the properties that were not different, helping to contribute to the body of knowledge on opportunities to improve the physical and functional properties of plant-based yogurts. By examining the impact of various ingredients on yogurt properties and conducting tribological analyses, we lay the foundation for developing more commercially viable plant-based yogurt formulations. Addressing the limitations of modern plant-based alternatives holds promise for enticing more individuals to embrace sustainable dietary choices, thus fostering a more environmentally conscious consumer base.

## Figures and Tables

**Table 1:** The mean and standard deviation of the nutritional content of dairy and plant-based yogurts reported per 100 g.

Base (n)	Energy (Kcal)	Fat (g)	Protein (g)	Carbohydrates (g)
Dairy	86.0 ± 19.0 ^a^	3.9 ± 0.7 ^a^	3.6 ± 0.6 ^a^	9.0 ± 3.8 ^a^
Soy *	93.3 ^ab^	2.3 ^a^	4.0 ^a^	14.0 ^a^
Oat	97.4 ± 13.0 ^ab^	3.5 ± 2.5 ^a^	3.0 ± 1.5 ^a^	12.8 ± 0.1 ^a^
Almond	101.6 ± 17.5 ^b^	5.5 ± 1.6 ^a^	2.3 ± 0.8 ^a^	11.5 ± 2.3 ^a^
Cashew	83.3 ± 14.1 ^ab^	4.3 ± 0.4 ^a^	2.0 ± 0.0 ^ab^	9.3 ± 4.7 ^a^
Coconut	76.8 ± 33.9 ^ab^	4.0 ± 2.0 ^a^	0.2 ± 0.3 ^b^	9.5 ± 5.2 ^a^

Columns report mean ± SD (standard deviation) for each nutrient, with significant differences within a column reported by different superscript letters (a, b) (*p* < 0.05). * For soy, only 1 product was tested; therefore, no SD was calculated.

**Table 2:** The mean and standard deviation of the pH and titratable acidity of dairy and plant-based yogurts.

Base (n)	pH	Lactic Acid %
Dairy	4.2 ± 0.2 ^ab^	0.9 ± 0.2 ^a^
Soy	4.8 ± 0.0 ^a^	0.8 ± 0.0 ^ab^
Almond	4.6 ± 0.2 ^a^	0.5 ± 0.1 ^ab^
Coconut	4.2 ± 0.1 ^ab^	0.3 ± 0.0 ^b^
Cashew	3.9 ± 0.2 ^b^	0.7 ± 0.1 ^ab^
Oat	3.7 ± 0.2 ^b^	0.3 ± 0.1 ^b^

Columns report mean ± SD for pH and lactic acid%. Significant differences within a column are reported using different superscript letters (a, b) (*p* < 0.05).

**Table 3:** The mean and standard deviation of the color of dairy and plant-based yogurts.

Base	L*	a*	b*	Whiteness Index
Dairy	91.9 ± 1.5 ^a^	−1.1 ± 0.3 ^a^	10.8 ± 0.9 ^ab^	86.5 ± 0.9 ^a^
Soy	87.2 ± 0.1 ^ab^	−0.5 ± 0.1 ^ab^	17.3 ± 0.1 ^bc^	78.5 ± 0.1 ^b^
Cashew	86.0 ± 0.7 ^ab^	0.2 ± 0.1 ^ab^	10.9 ± 0.7 ^abc^	82.3 ± 0.5 ^c^
Coconut	85.8 ± 3.0 ^b^	−0.1 ± 0.6 ^ab^	7.5 ± 3.8 ^a^	83.9 ± 2.5 ^abc^
Oat	78.2 ± 0.3 ^b^	1.6 ± 1.0 ^b^	17.0 ± 1.8 ^c^	72.3 ± 1.0
Almond	78.4 ± 3.8 ^b^	0.9 ± 1.1 ^b^	10.1 ± 2.0 ^abc^	76.1 ± 2.3 ^b^

Mean ± SD with significant differences between columns reported using different superscript letters (a, b, c) in the same column (*p* < 0.05). The Whiteness Index was calculated using the equation mentioned in the methods.

**Table 4:** The mean and standard deviation of flow behavior index (n), consistency coefficient (k), R2, apparent viscosity at the shear rate 25 (ƞapp25), and area in the loop (HLA) of plant-based and dairy yogurts.

Base	n	K	R^2^	ƞapp25	HLA
Dairy	0.07 ± 0.1	70.6 ± 39.5	0.38 ± 0.4	3.3 ± 1.4	6821.1 ± 73.9 ^a^
Coconut	0.26 ± 0.1	25.9 ± 9.8	0.94 ± 0.0	2.7 ± 1.9	3428.3 ± 99.3 ^b^
Almond	0.18 ± 0.0	39.0 ± 14.6	0.86 ± 0.0	2.8 ± 0.9	3416.4 ± 78.3 ^c^
Cashew	0.25 ± 0.1	38.6 ± 24.0	0.81 ± 0.1	2.9 ± 0.7	2966.5 ± 92.0 ^d^
Oat	0.37 ± 0.1	17.4 ± 15.0	0.94 ± 0.0	1.6 ± 0.8	2853.3 ± 56.5 ^e^
Soy	0.21 ± 0.0	33.3 ± 1.3	0.98 ± 0.0	2.6 ± 0.0	2051.7 ± 83.1 ^f^

Columns report mean ± SD for each rheological parameter. Significant differences within a column are reported using different superscript letters (a to f) in the same column (*p* < 0.05).

## Data Availability

The original contributions presented in the study are included in the article/supplementary material; further inquiries can be directed to the corresponding author.

## Supplementary Materials

The following are available online at https://www.mdpi.com/article/10.3390/foods13070984/s1, Table S1: A list of products and their ingredients.
