# Supplementary material for: Comparison of Physical and Compositional Attributes between Commercial Plant-Based and Dairy Yogurts
Source: Foods. 2024 Mar 23;13(7):984. doi: 10.3390/foods13070984 (PMC11011924; doi:10.3390/foods13070984)
Supplement: Supplementary file 1 [file foods-13-00984-s001.zip › foods-2917027-supplementary.pdf]

Supplemental Table S1

S.No	Brand	Base	Flavor	Ingredients
1	Oatly	Oat	Plain	Oatmilk (Water, Oats), Low Erucic Acid Rapeseed Oil, Potato Starch. Contains 2% Or Less Of: Dextrose, Pea Protein, Potato Protein, Calcium Carbonate, Guar Gum, Tricalcium Phosphate, Locust Bean Gum, Live Active Cultures (*S. Thermophilus, L. Bulgaricus, L. Casei, L. Acidophilus,* and *B. Lactis*).
2	Kite Hill	Almond	Vanilla	Almond Milk (Water, Almonds), Cane Sugar, Starch, Citrus Fiber, Natural Flavors, Vanilla Extract, Locust Bean Gum, Citric Acid, Xanthan Gum, Vanilla Bean Seeds, Live Active Cultures
3	Kite Hill	Almond	Plain	Almond Milk (Water, Almonds), Locust Bean Gum, Xanthan Gum, Agar, Live Active Cultures.
4	Oui	Coconut	Vanilla	Coconut Base (Water, Coconut Cream, Cane Sugar, Modified Food Starch, Natural Flavor, Tricalcium Citrate, Malic Acid, Vanilla Extract, Vitamin D2), Cultures.
5	Silk	Almond	Vanilla	Almond Milk (Water, Almonds), Cane Sugar, Water, Pectin, Natural Flavors, Calcium Citrate, Calcium Phosphate, Citric Acid, Vanilla Bean Specks, Live And Active Cultures, Vitamin D2. Live And Active Cultures: *S. Thermophilus, L. Bulgaricus, L. Acidophilus, B. Lactis*.
6	Silk	Soy	Vanilla	Soymilk (Water; Soybeans), Cane Sugar, Corn Starch, Pectin, Tricalcium Phosphate, Citric Acid, Natural Flavor, Dipotassium Phosphate, Sea Salt, Vanilla Bean, Live and Active Cultures, Mixed Tocopherols and Vitamin C Ester (To Protect Freshness), Vitamin D2.
7	So Delicious	Coconut	Unsweetened Vanilla	Organic Coconut Milk (filtered Water, Organic Coconut Cream), Vitamin And Mineral Blend (calcium Phosphate, Magnesium Phosphate, Calcium Carbonate, L-selenomethionine [selenium], Vitamin A Acetate, Vitamin D2, Zinc Oxide, Vitamin B12), Organic Sunflower Lecithin, I Gellan Gum, Natural Flavor, Organic Locust Bean Gum, Sea Salt.
8	So Delicious	Coconut	Vanilla	Organic Coconut Milk (Filtered Water, Organic Coconut Cream), Organic Cane Sugar, Rice Starch, Contains 2% Or Less Of: Natural Flavor, Calcium Citrate, Pectin, Locust Bean Gum, Citric Acid, Live And Active Cultures, Vitamin D2, Vitamin B12.
9	So Delicious	Coconut	Unsweetened Plain	Organic Coconut Milk (Filtered Water; Organic Coconut Cream), Rice Starch, Contains 2% or Less of: Pectin, Calcium Phosphate, Dipotassium Phosphate, Live and Active Cultures, Locust Bean Gum, Vitamin D2, Vitamin B12
10	Forager	Cashew	Vanilla Bean	Cashew Milk (Filtered Water, Cashews*), Cane Sugar*, Tapioca Starch*, Pectin, Locust Bean Gum*, Natural

				Flavor*, Vanilla Beans*, Lemon Juice Concentrate*, Coconut Cream*, Live Active Cultures. (*ORGANIC). Live Active Cultures: *S. Thermophilus, L. Bulgaricus, L. Acidophilus, Bifidus, L. Lactis, L. Plantarum.*
11	Forager	Cashew	Plain	Cashew milk (filtered Water, Cashews*), Tapioca Starch*, Locust Bean Gum*, Coconut Cream*, Live Active Cultures.
12	Almond Breeze	Almond	Vanilla	Almond milk (filtered Water, Almonds), Sugar, Tapioca Starch, Pectin, Natural Flavors, Vanilla Extract, Agar, Calcium Phosphate, Salt, Locust Bean Gum, Sodium Citrate, Calcium Lactate and Cultures.
13	Chobani	Oat	Vanilla	Oat Blend* (Water, Whole Grain Oats*), Cane Sugar*, Water, Pea Protein*, Tapioca Flour, Less Than 2% Of: Coconut*, Vanilla Extract, Sunflower Oil*, Natural Flavors, Fruit Pectin, Lemon Juice Concentrate*, Locust Bean Gum*, Cultures, *Organic Ingredients
14	Brown Cow	Whole Milk	Plain	Cultured Pasteurized Whole Milk, Pectin
15	Brown Cow	Whole Milk	Vanilla	Cultured Pasteurized Whole Milk, Pectin
16	Dannon	Whole Milk	Plain	Cultured Grade A Milk
17	Noosa	Whole Milk	Vanilla	Grade A Pasteurized Whole Milk, Cane Sugar, Vanilla Blend (Cane Sugar, Water, Tapioca Starch, Vanilla Extract, Vanilla Bean, Natural Flavor, Pectin, Lemon Juice Concentrate), Wildflower Honey, Kosher Gelatin, Pectin, Live Active Cultures. Live Active Cultures: *S. Thermophilus, L. Bulgaricus, L. Acidophilus, Bifidus, L. Casei.*
18	Stony Field	Whole Milk	Plain	Cultured Pasteurized Organic Whole Milk, Pectin, Vitamin D3. 6 Live Active Cultures: *S. Thermophilus, L. Bulgaricus, L. Acidophilus, Bifidus, L. Paracasei and L. Rhamnosus*.
19	Stony Field	Whole Milk	Vanilla	Cultured Pasteurized Organic Whole Milk, Organic Cane Sugar, Organic Natural Vanilla Flavor, Pectin, Lactase Enzyme, Vitamin D3
20	Nature's Promise	Whole Milk	Plain	Cultured Pasteurized Grade A Organic Milk, Pectin, Vitamin D3 Added. Live & Active Cultures: *L. Acidophilus, Bifidus, L.casei*.
21	365	Whole Milk	Vanilla	Cultured Pasteurized Organic Whole Milk, Organic Cane Sugar, Organic Natural Vanilla Flavor, Pectin, Vitamin D3, Contains Live and Active Cultures: *S. Thermophilus, L. Bulgaricus, L. Acidophilus, Bifidus, L. Paracasei, L. Rhamnosus*
